# Supplementary material for: Hot spot prediction in protein-protein interactions by an ensemble system
Source: BMC Syst Biol. 2018 Dec 31;12(Suppl 9):132. doi: 10.1186/s12918-018-0665-8 (PMC6311905; doi:10.1186/s12918-018-0665-8)
Supplement: Supplementary file 2 — All relevant MATLAB code. A simple Matlab implement of our predictor. (ZIP 19,742 kb) [file 12918_2018_665_MOESM2_ESM.zip › Additional file 2/Read ASA/ASA website/dbMPIKT/1CZ8W.html]

 NetSurfP - Protein Surface Accessibility and Secondary Structure Predictions

  

|  |  |  |
| --- | --- | --- |
|  |  | NetSurfP - Protein Surface Accessibility and Secondary Structure Predictions  Technical University of Denmark |

  


---

```
# For publication of results, please cite:
# A generic method for assignment of reliability scores applied to solvent accessibility predictions.
# Bent Petersen, Thomas Nordahl Petersen, Pernille Andersen, Morten Nielsen and Claus Lundegaard
# BMC Structural Biology 2009, 9:51 doi:10.1186/1472-6807-9-51
#
# Column 1: Class assignment - B for buried or E for Exposed - Threshold: 25% exposure, but not based on RSA
# Column 2: Amino acid
# Column 3: Sequence name
# Column 4: Amino acid number
# Column 5: Relative Surface Accessibility - RSA
# Column 6: Absolute Surface Accessibility
# Column 7: Z-fit score for RSA prediction
# Column 8: Probability for Alpha-Helix
# Column 9: Probability for Beta-strand
# Column 10: Probability for Coil
E V  1CZ8W                  1    0.842 129.369  -0.693   0.003   0.003   0.994
B V  1CZ8W                  2    0.271  41.730   0.010   0.053   0.043   0.903
E K  1CZ8W                  3    0.474  97.584   0.678   0.184   0.043   0.773
B F  1CZ8W                  4    0.220  44.174  -1.051   0.430   0.016   0.555
E M  1CZ8W                  5    0.373  74.677  -0.438   0.660   0.049   0.291
E D  1CZ8W                  6    0.520  74.889  -0.081   0.605   0.105   0.290
B V  1CZ8W                  7    0.242  37.195  -0.289   0.649   0.163   0.188
B Y  1CZ8W                  8    0.253  54.109   0.024   0.502   0.102   0.396
E Q  1CZ8W                  9    0.553  98.837   0.619   0.278   0.093   0.628
E R  1CZ8W                 10    0.574 131.332   0.328   0.113   0.043   0.844
B S  1CZ8W                 11    0.156  18.236  -2.303   0.052   0.084   0.864
E Y  1CZ8W                 12    0.473 101.016   0.574   0.021   0.279   0.699
B C  1CZ8W                 13    0.045   6.388  -1.752   0.022   0.359   0.619
E H  1CZ8W                 14    0.420  76.380   1.040   0.004   0.420   0.576
E P  1CZ8W                 15    0.352  49.977  -0.513   0.005   0.336   0.660
E I  1CZ8W                 16    0.370  68.376   0.790   0.005   0.336   0.660
E E  1CZ8W                 17    0.594 103.702  -1.068   0.004   0.514   0.481
E T  1CZ8W                 18    0.318  44.079   0.655   0.001   0.900   0.099
B L  1CZ8W                 19    0.226  41.472   0.280   0.001   0.959   0.040
B V  1CZ8W                 20    0.070  10.713   1.006   0.001   0.959   0.040
E D  1CZ8W                 21    0.408  58.807   0.598   0.001   0.959   0.040
B I  1CZ8W                 22    0.044   8.085   0.137   0.021   0.756   0.223
E F  1CZ8W                 23    0.297  59.628   0.336   0.003   0.718   0.279
E Q  1CZ8W                 24    0.525  93.711  -0.338   0.022   0.552   0.426
E E  1CZ8W                 25    0.561  97.972  -0.439   0.022   0.359   0.619
E Y  1CZ8W                 26    0.311  66.354  -0.328   0.018   0.088   0.893
E P  1CZ8W                 27    0.459  65.061  -1.337   0.018   0.047   0.935
E D  1CZ8W                 28    0.651  93.838  -0.756   0.018   0.047   0.935
E E  1CZ8W                 29    0.484  84.485  -0.538   0.018   0.047   0.935
B I  1CZ8W                 30    0.229  42.458  -0.470   0.019   0.141   0.840
B E  1CZ8W                 31    0.304  53.179  -0.471   0.005   0.336   0.660
B Y  1CZ8W                 32    0.156  33.401   0.127   0.004   0.616   0.381
B I  1CZ8W                 33    0.184  33.985   0.328   0.001   0.900   0.099
B F  1CZ8W                 34    0.062  12.363  -0.146   0.001   0.900   0.099
B K  1CZ8W                 35    0.218  44.945   0.947   0.004   0.616   0.381
B P  1CZ8W                 36    0.063   8.897  -0.364   0.004   0.197   0.799
B S  1CZ8W                 37    0.265  31.081  -0.632   0.004   0.138   0.858
B C  1CZ8W                 38    0.064   8.915  -0.340   0.004   0.514   0.481
B V  1CZ8W                 39    0.077  11.835   0.351   0.018   0.846   0.136
E P  1CZ8W                 40    0.265  37.575   0.582   0.011   0.918   0.071
B L  1CZ8W                 41    0.052   9.594   0.444   0.018   0.846   0.136
B M  1CZ8W                 42    0.241  48.244   0.504   0.021   0.756   0.223
B R  1CZ8W                 43    0.233  53.357  -0.580   0.023   0.655   0.322
B C  1CZ8W                 44    0.033   4.605  -1.406   0.021   0.451   0.528
B G  1CZ8W                 45    0.156  12.309  -1.731   0.021   0.279   0.699
B G  1CZ8W                 46    0.118   9.263  -3.247   0.004   0.197   0.799
B C  1CZ8W                 47    0.098  13.703  -0.737   0.020   0.205   0.775
B C  1CZ8W                 48    0.053   7.511   0.052   0.019   0.141   0.840
E N  1CZ8W                 49    0.374  54.812  -0.586   0.018   0.047   0.935
E D  1CZ8W                 50    0.480  69.110  -1.554   0.018   0.019   0.964
E E  1CZ8W                 51    0.672 117.381  -0.227   0.018   0.047   0.935
E G  1CZ8W                 52    0.512  40.294  -0.182   0.004   0.085   0.910
B L  1CZ8W                 53    0.176  32.299   0.657   0.004   0.420   0.576
E E  1CZ8W                 54    0.438  76.519   0.825   0.002   0.816   0.182
B C  1CZ8W                 55    0.052   7.273  -1.700   0.001   0.900   0.099
E V  1CZ8W                 56    0.343  52.781   0.954   0.002   0.816   0.182
B P  1CZ8W                 57    0.235  33.417  -1.364   0.004   0.616   0.381
E T  1CZ8W                 58    0.373  51.693  -0.168   0.004   0.514   0.481
E E  1CZ8W                 59    0.540  94.355  -0.020   0.003   0.718   0.279
E E  1CZ8W                 60    0.499  87.245  -0.019   0.002   0.816   0.182
E S  1CZ8W                 61    0.325  38.149   0.333   0.001   0.900   0.099
E N  1CZ8W                 62    0.522  76.465   0.028   0.001   0.900   0.099
B I  1CZ8W                 63    0.181  33.559   1.064   0.001   0.959   0.040
E T  1CZ8W                 64    0.367  50.889   0.581   0.001   0.959   0.040
B M  1CZ8W                 65    0.098  19.610   1.030   0.001   0.959   0.040
E Q  1CZ8W                 66    0.294  52.491   1.325   0.001   0.959   0.040
B I  1CZ8W                 67    0.047   8.695  -0.436   0.001   0.959   0.040
B M  1CZ8W                 68    0.233  46.723  -0.177   0.001   0.959   0.040
E R  1CZ8W                 69    0.298  68.242  -0.107   0.001   0.900   0.099
B I  1CZ8W                 70    0.105  19.370  -0.171   0.002   0.816   0.182
E K  1CZ8W                 71    0.510 104.989   0.354   0.004   0.616   0.381
E P  1CZ8W                 72    0.331  46.912  -0.175   0.005   0.336   0.660
E H  1CZ8W                 73    0.597 108.540  -1.814   0.004   0.138   0.858
E Q  1CZ8W                 74    0.499  89.050  -0.945   0.004   0.138   0.858
E G  1CZ8W                 75    0.593  46.677  -0.617   0.005   0.262   0.733
E Q  1CZ8W                 76    0.564 100.748   0.096   0.004   0.197   0.799
E H  1CZ8W                 77    0.535  97.317  -0.529   0.004   0.420   0.576
E I  1CZ8W                 78    0.423  78.329  -0.093   0.002   0.816   0.182
B G  1CZ8W                 79    0.206  16.204   0.454   0.001   0.900   0.099
E E  1CZ8W                 80    0.518  90.530   1.124   0.001   0.959   0.040
B M  1CZ8W                 81    0.064  12.826   1.025   0.001   0.900   0.099
E S  1CZ8W                 82    0.353  41.383   1.426   0.002   0.816   0.182
B F  1CZ8W                 83    0.095  19.147   0.685   0.021   0.756   0.223
E L  1CZ8W                 84    0.493  90.268   0.629   0.023   0.655   0.322
E Q  1CZ8W                 85    0.512  91.372   0.326   0.022   0.552   0.426
E H  1CZ8W                 86    0.229  41.691   0.183   0.022   0.359   0.619
E N  1CZ8W                 87    0.557  81.486   0.465   0.022   0.359   0.619
E K  1CZ8W                 88    0.629 129.344   1.779   0.004   0.616   0.381
B C  1CZ8W                 89    0.084  11.864  -0.375   0.003   0.718   0.279
E E  1CZ8W                 90    0.558  97.465   1.370   0.002   0.816   0.182
B C  1CZ8W                 91    0.089  12.453  -2.125   0.021   0.756   0.223
E R  1CZ8W                 92    0.537 123.019   1.409   0.004   0.616   0.381
E P  1CZ8W                 93    0.561  79.648  -1.587   0.005   0.336   0.660
E K  1CZ8W                 94    0.788 162.194   0.842   0.003   0.003   0.994

Explain the output.  Go back.
```

---
